# Artificial Intelligence in Cervical Cytology: Opportunities and Limitations in Screening, Triage, and Diagnostic Support

**DOI:** 10.3390/diagnostics16101541

**Published:** 2026-05-19

**Authors:** Agata Stanek-Widera, Jędrzej Borowczak, Dominik Skiba, Michel-Edwar Mickael, Marzena Łazarczyk, Mateusz Maniewski, Łukasz Szylberg, Andrey Bychkov, Piotr Religa

**Affiliations:** 1 Faculty of Medicine, Academy of Silesia, 40-555 Katowice, Poland; aswidera502@gmail.com; 2 Faculty of Medicine, Bydgoszcz University of Science and Technology, Aleje Prof. S. Kaliskiego 7, 85-796 Bydgoszcz, Poland; 3 Institute of Genetics and Animal Biotechnology of the Polish Academy of Sciences, 05-552 Magdalenka, Poland; 4 Department of Obstetrics, Gynaecology and Oncology, Collegium Medicum, Nicolaus Copernicus University in Bydgoszcz, 85-067 Bydgoszcz, Poland; 5 Department of Pathology, Kameda Medical Center, Kamogawa 296-8602, Chiba, Japan; bychkov.andrey@kameda.jp; 6 Department of Medicine Solna, Karolinska Institute, 171 77 Stockholm, Sweden

**Keywords:** artificial intelligence, cytology, digital diagnosis

## Abstract

Cervical cancer remains a major global health challenge, particularly in low- and middle-income countries, where access to screening, vaccination, and timely treatment may be limited. Cervical cytology has played an important historical role in prevention, but it is labor-intensive, time-consuming, and subject to observer variability and limited sensitivity. In many contemporary screening programs, HPV testing is now used as the primary screening test, while cytology is used mainly for the triage of HPV-positive women. In recent years, artificial intelligence (AI), particularly deep learning (DL), has shown considerable potential in medical image analysis and computer-aided diagnosis. This review summarizes current applications of AI in cervical cytology and related diagnostic workflows, including automated and assisted slide screening, liquid-based cytology, the triage of equivocal or HPV-positive cases, and colposcopy support. Across these settings, AI-assisted systems may improve efficiency, standardization, and diagnostic consistency, and may reduce workload in resource-constrained environments. However, the evidence is heterogeneous, and important challenges remain, including the need for large and diverse datasets, prospective validation, regulatory approval, digital infrastructure, workflow integration, and the resolution of ethical and legal issues. AI should therefore be regarded as a promising adjunct to human expertise rather than a replacement in cervical cytology and related clinical diagnostic pathways.

## 1. Introduction

Cervical cancer poses a serious threat to women’s health worldwide, being the fourth-most frequently diagnosed cancer and the fourth leading cause of cancer-related death in women, accounting for approximately 600,000 new cases and over 300,000 deaths annually worldwide. In 37 countries, with 29 of those countries in sub-Saharan Africa and the rest in Central and South America, cervical cancer is actually the leading cause of cancer death in women due to limited screening and HPV vaccination access [1,2]. Early screening and diagnosis are fundamental strategies proven to reduce both the incidence and mortality rates of cervical cancer [3,4]. In contemporary screening programs, HPV testing has increasingly replaced cytology as the primary screening test, and the World Health Organization recommends HPV testing as the preferred primary screening method [5]. In this setting, cytology is used mainly as a triage tool for women with a positive HPV test rather than as the primary screening method itself. Nevertheless, cervical cytology remains widely used in many regions and continues to play an important role in both screening and triage workflows [6,7].

For decades, cervical cytology, often involving microscopic analysis of Pap smears, has served as a primary screening tool for identifying precancerous and cancerous cells [8,9]. However, the manual review of cervical cytology slides is labor-intensive and time-consuming. A single slide can contain thousands of cells, requiring meticulous analysis by expert cytologists and pathologists [10,11]. Detecting morphological changes in these cells is challenging and highly dependent on the reviewer’s experience. These factors contribute to diagnostic variability and can result in relatively high false-positive and false-negative rates [12,13]. Furthermore, the manual process requires significant human resources, and some regions, such as China, face challenges due to an insufficient number of professionals trained to read the vast number of slides generated by screening programs [10].

The advent of and rapid advancements in artificial intelligence (AI), particularly in the domain of medical image analysis, offer promising solutions to the limitations of traditional cytology [14,15]. AI applications have already begun to transform various aspects of daily life, including areas like speech recognition and robotics [16]. In medicine, the potential of computers was recognized early on, with claims dating back to 1959 suggesting that diagnostic interpretations could be carried out by machines. By the 1970s, predictions were made that computing science would significantly impact medicine, potentially augmenting and replacing some intellectual functions of the physician [17].

AI has seen remarkable growth in healthcare over the past ten years [18]. Its capacity to evaluate and interpret image data has made significant progress, spurred by recent advancements in computer vision and machine learning [19]. One of the first applications of AI in a pathology laboratory was in cytopathology, specifically with the creation of a computer-assisted Pap test screening system. While initially designed for diagnosis rather than screening, these early systems faced disagreement regarding their widespread clinical use [19,20].

Today, AI-based medical diagnostic applications are increasingly being applied in the screening and diagnosis of cervical cancer [21]. The reported advantages include shorter analysis times, reduced reliance on highly trained personnel, and the minimization of observer bias [22]. AI is expected to serve as a practical tool for doctors, aiding in accurate clinical diagnoses. A Delphi study of experts in computational pathology predicted that cervical cytology diagnosis in laboratories may be largely outsourced to AI by 2030 [22,23], assuming the continued expansion and maturation of laboratory digitization infrastructures [24]. This review provides an overview of the current applications, diagnostic performance, and limitations of AI technology in cervical cytology, drawing upon findings from various studies and reviews presented in published studies and systematic reviews. This article is a narrative review aimed at providing an overview of current applications and challenges of artificial intelligence in cervical cytology. The literature was selected based on the authors’ expertise and a targeted search of major databases (e.g., PubMed, Scopus, Web of Science) focusing on recent and clinically relevant studies.

## 2. Artificial Intelligence Techniques in Cervical Cytology

AI, including machine learning (ML) and its subfield deep learning (DL), is increasingly being used in medical image analysis. In cytopathology, DL algorithms have contributed significantly to advances in feature extraction and image classification, becoming a key area of research and development [19].

Various ML and DL techniques have been applied to the analysis of cervical cytology images, including classic ML models such as k-nearest neighbors [25], artificial neural networks [26], decision trees [27,28], random forests [29], support vector machines [30], and logistic regression [28]. Deep learning models, particularly convolutional neural networks (CNNs) [31,32,33], multilayer perceptrons [34], deep neural networks [35], and architectures such as residual networks (ResNet) [36] and Inception V3 [37], have shown strong performance in image-based tasks. Techniques like the Synthetic Minority Oversampling Technique (SMOTE) have also been employed, often in combination with other algorithms [38,39].

In computational cytology, DL-based approaches are being explored for various tasks, including the comprehensive analysis of digitized cytology images for cancer screening. Different DL schemes have been employed, including fully supervised, weakly supervised, unsupervised, and transfer learning [40]. The last, for instance, involves using knowledge gained from training on a large dataset for a source task and applying it to a related but different target task, which can be particularly useful when data for the specific cytology problem is lacking [31].

A key challenge in automated cytology image analysis is accurately processing cellular details, especially in slides with clustered or overlapping cells. To overcome these issues, DL models have been developed to segment cytological images into discrete components, such as individual nuclei and surrounding cytoplasm, by refining cell boundaries and resolving overlaps [41]. Several architectures have been applied to support cervical lesion classification, including approaches combining small-object detection generative adversarial networks and fine-tuned stacked autoencoders, which process nuclei and cytoplasm independently [42,43]. Beyond segmentation, AI algorithms facilitate feature extraction and autonomous data processing for downstream classification tasks [42]. DL models can also extract feature fusion vectors from pre-trained models, which can then be used for classification through various workflows, including those involving principal component analysis and ML ensembles, or artificial neural networks [44]. These approaches aim to replicate the visual interpretation typically performed by cytologists—isolating diagnostically relevant regions and assigning lesion categories—with reasonable consistency and scalability.

The development of whole-slide imaging (WSI) technology, which enables the digitization of entire cytology slides into high-resolution images, has been the cornerstone of implementing AI-based automated image analysis in cytology [45]. These systems can process WSIs at multiple levels, detecting cellular abnormalities within localized fields and producing overall slide-level predictions [46,47,48]. This allows for the development of models capable of both highlighting individual abnormal cells and classifying the entire slide as neoplastic or non-neoplastic [49].

Whole-slide imaging in cytology presents unique technical challenges compared with histology, primarily due to the three-dimensional nature of cytology preparations. Unlike thin tissue sections, cytology specimens contain cells and clusters distributed across multiple focal planes, requiring specialized scanning strategies to capture diagnostic cellular detail throughout the specimen’s depth. Single-layer scanning acquires one focal plane per tile using autofocus algorithms that target cellular features, offering fast scan times and manageable file sizes but potentially missing cells outside the chosen plane [50,51]. Multilayer or Z-stack acquisition addresses this limitation by capturing multiple focal planes at defined micron intervals, allowing reviewers to navigate through the z-axis to bring all cells into focus; however, this approach substantially increases both scanning time and file size [50,52]. Extended depth of field (EDOF) fusion represents an alternative strategy that computationally combines information from multiple focal planes into a single composite image in which objects at different depths appear in focus, thereby reducing the need for z-navigation while maintaining the visibility of cellular details in thick clusters [53,54,55,56,57,58]. Some implementations employ hardware-assisted methods such as slanted-stage scanning to acquire multi-depth data efficiently and apply fusion algorithms to generate all-in-focus outputs [59,60]. The choice among these approaches depends on specimen characteristics, workflow requirements, and the balance between scanning throughput, file size, and diagnostic coverage, with comparative studies showing that the scanner selection, cytopreparation method, and focus settings significantly impact the capture of atypical cells and practical scanning performance [21,51,61].

AI algorithms can also be trained based on specific cytological features. For example, in studies applying AI to urine cytology based on The Paris System, features such as the nucleus-to-cytoplasm (N:C) ratio and chromatin patterns like hyperchromasia were used as input to DL models [62]. Iterative processes involving reviewer feedback can be used to improve intermediate algorithms and accumulate additional training data [49].

## 3. Applications of AI in Cervical Cytology Screening and Diagnosis

AI is being applied across various stages of the cervical cancer screening and diagnostic pathway, aiming to improve efficiency, accuracy, and access (Table 1).

### 3.1. Automated and Assisted Screening Systems

The primary application of AI in cervical cytology has been its incorporation into automated or computer-assisted screening systems [19]. The goal is to support cytotechnologists—trained specialists who prepare and examine biological specimens under the supervision of cytopathologists—and assist cytopathologists in slide review and final diagnosis, either by automatically classifying slides or by highlighting potentially abnormal areas or cells for manual review [69]. Early computer-assisted screening systems focused on processing slides and identifying regions of interest (ROIs) for cytotechnologists to examine, requiring full manual review only if abnormalities were detected. More recent systems incorporate advanced AI algorithms to improve efficiency and diagnostic accuracy [14].

**Figure 1:**
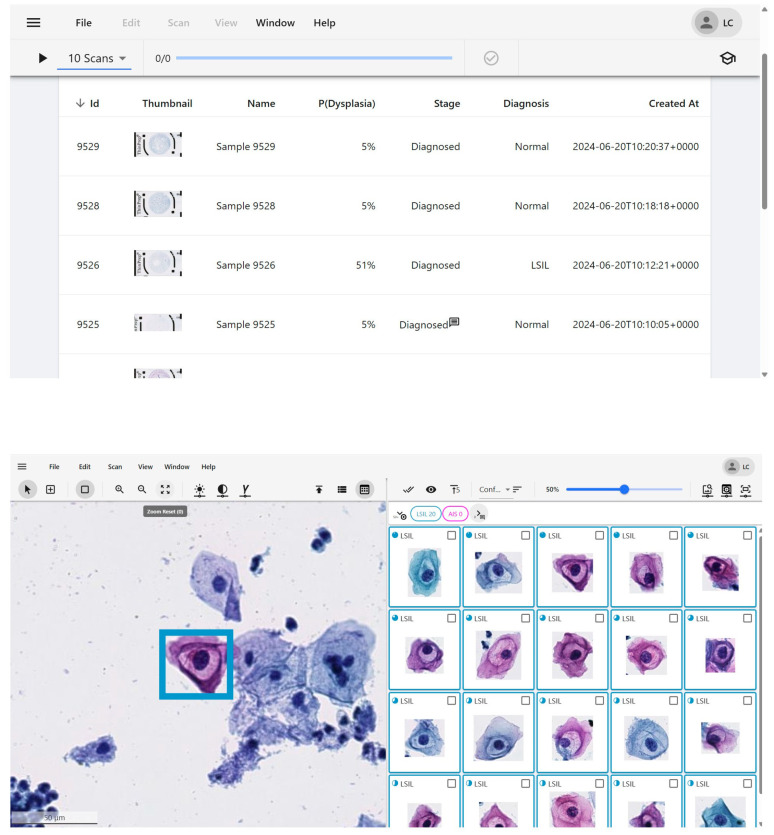
Example of the user interface of a cervical cytology AI system (Scanome Ltd., Warsaw, Poland). Results are displayed in the ScanAI Viewer, which presents detected dysplastic cells ranked by probability of dysplasia. Outputs support diagnostic review, case prioritization, and quality control across participating centers. [Disclosure: Scanome Ltd. is a commercial product. Piotr Religa is the owner of Scanome Ltd. This figure is included solely for illustrative purposes].

There are several commercially available, FDA-approved, and CE-marked computer-assisted screening systems for Pap tests [14]. Examples include the ThinPrep Imaging System (TIS), which has been upgraded with an AI algorithm in the CE-marked, Genius Digital Diagnostics System—the only FDA-cleared system—and the BD FocalPoint GS imaging system [70]. The BD FocalPoint Slide Profiler, which received FDA approval, can be applied to both liquid-based cytology and conventional smears and classifies slides by risk level, with some labeled “no further review” if the risk is low [71,72]. Other CE-marked systems include CytoProcessor by DATEXIM (Caen, France), a fully digital virtual slide presentation system that uses AI-based abnormal-cell identification to support remote diagnostic workflows. In a comparative study using a consensus cytology reference standard in an enriched study set, CytoProcessor showed fewer undercalled cytologic abnormalities than the ThinPrep Imaging System (1.5% vs. 4.0%) and reduced worker time, although the total time per slide was similar when machine time was included [73]. The Techcyte SureView Cervical Cytology System, developed in collaboration with BD, is a CE-marked AI-assisted screening tool that aids cytotechnologists and pathologists in analyzing diagnostically important cells and organisms in SurePath and ThinPrep slides using an AI-based algorithm with workflow and LIS integration; it has been clinically deployed at Tyrolpath in Austria as the first clinical adoption in Europe [Techcyte]. Landing AI’s cervical screening system, certified to perform cytological and histopathological diagnosis, features fully automated scanners with an online artificial intelligence assessment system and has been used in large-scale screening programs in China and other regions in Europe and Asia [73,74]. Additionally, Holmström et al. used the commercially available cloud-based image-analysis platform Aiforia Create to develop a deep learning system for point-of-care cervical cytology screening in a resource-limited setting. In that proof-of-concept study, the system achieved high sensitivity for detecting cervical cellular atypia (95.7–100%) with AUC values of 0.94–0.96, depending on the reference standard used [75]. These systems can automatically identify abnormal cells for confirmation by cytotechnologists, with their primary function being to identify abnormal cells and differentiate negative from positive slides (Figure 1), thereby assisting professionals in improving screening accuracy and efficiency [14,21].

In a multicenter, clinic-based observational study of referral women, AI-assisted reading detected 92.6% of CIN2 and 96.1% of CIN3+ lesions. Compared with skilled cytologists, AI-assisted reading showed equivalent sensitivity for CIN2+ and higher specificity, suggesting potential value in cervical cytology screening and triage [22]. Another study comparing AI-assisted reading with four pathologists demonstrated that the AI model achieved a high area under the receiver operating characteristic curve (AUC) of 0.99 and high accuracy [21,76].

AI microscopes equipped with augmented reality (AR) techniques represent another approach, providing real-time onsite diagnostic assistance by projecting the results of AI algorithms onto the observer’s field of view [76]. A preliminary study evaluating such an AI microscope showed improvements in sensitivity for detecting low-grade squamous intraepithelial lesion (LSIL+)-positive cases and “atypical squamous cells—cannot exclude high-grade squamous intraepithelial lesion” (ASC-H) cases [10]. The study also reported that AI microscopes improved the intraobserver agreement among cytopathologists for binary classification (from 0.649 to 0.706) and multiclassification, and for atypical squamous cells of undetermined significance (ASC-US) diagnoses. These findings suggest that AI microscope assistance can improve the efficiency and accuracy of cervical cytology diagnosis and enhance diagnostic consistency [10]. Liquid-based cytology (LBC) has become more common than conventional smears for cervical cancer screening [45]. When LBC specimens are digitized into WSIs, they enable AI-based automated image analysis. AI models using DL have been investigated for classifying WSIs of LBC specimens into neoplastic and non-neoplastic categories. Several pilot studies using large training and test sets have demonstrated good classification performance on WSIs, with one of them achieving AUC values ranging from 0.89 to 0.96 across different test sets [45]. DL-based hybrid methodologies have also been proposed for classifying Pap smear cytology images, including those prepared using LBC [44]. Evaluation of these methods on public benchmark datasets, particularly those including multi-cell LBC samples, has shown promising results [44]. However, cytological features in LBC can differ depending on the type of preservative solution used, which may impact the cell detection rates of AI algorithms [76]. This highlights a factor to consider in the development and application of AI for LBC.

#### AI-Supported Triage in ASC-US

AI is also being explored for its potential role in the triage of patients with equivocal cytology results. For instance, while most ASC-US cases (accounting for about 5% of Pap smears) are benign or low-grade abnormalities on histopathology, some may be precancerous or cancerous. Efficient risk stratification strategies for ASC-US are critical to reduce overtreatment, decrease patient anxiety, and save resources [64].

To address this need, an AI-based triage system has been developed and evaluated for predicting CIN2+ lesions from ASC-US cytology. This system uses a DL model that extracts both cell-level and slide-level information. In a study comparing this AI triage system to high-risk human papillomavirus (hrHPV) testing, the AI system demonstrated higher sensitivity (92.9% vs. 89.3%) and specificity (49.7% vs. 34.3%), without incurring additional costs. Given the high population prevalence of hrHPV and limited specificity, especially in younger populations, AI-based triage could offer a valuable alternative or complementary approach [64].

### 3.2. AI-Supported Triage in HPV-Based Screening

Beyond cytology, the use of AI algorithms has also been explored to support HPV-based screening strategies. Due to the high prevalence of HPV infections, triaging HPV-positive women to identify those at high risk of CIN2+ lesions remains a clinical and logistical challenge [67,77]. Conventional triage methods, such as cytology and HPV16/18 genotyping, are limited by poor reproducibility, high prices (in low-income countries), and high inter-observer disagreement. They also warrant a high number of colposcopy referrals and unnecessary procedures, putting additional burdens on healthcare systems, especially in low-resource settings [78].

Several studies have explored AI models to improve triage accuracy and efficiency in HPV-based screening workflows. One of the most comprehensive evaluations was conducted by Wentzensen et al., who developed a two-stage deep learning model to automatically interpret p16/Ki67 dual-stained liquid-based cytology (LBC) slides from HPV-positive women. In the SurePath validation study, the AI system showed high diagnostic performance for CIN3+ detection, with 88.1% sensitivity and 61.5% specificity, and reduced colposcopy referrals compared with Pap cytology (41.9% vs. 60.1%). The model was also shown to be robust across multiple scanning platforms and potentially applicable in vaccinated populations with declining disease prevalence [68].

Similarly, an AI-enabled LBC system was investigated for triaging HPV-positive women. AI-LBC showed comparable sensitivity to and significantly higher specificity than cytologists for detecting CIN2+, reduced colposcopy referrals by 9.4 percentage points, and had substantially higher sensitivity than HPV16/18 genotyping. Similar patterns were observed for CIN3+ [79]. In the subsequent multicenter evaluation, AI-assisted cytology improved triage performance among HPV-positive women, with higher sensitivity and specificity than manual cytology interpretation and fewer colposcopy referrals, particularly for junior cytopathologists [67]. These findings suggest that high-performance AI-supported cytology may improve the efficiency of colposcopy referral, but they do not support the replacement of colposcopy and biopsy as the diagnostic standard.

### 3.3. AI in Colposcopy

AI is also being applied in colposcopy, which is typically recommended following an abnormal Pap smear or positive high-risk HPV test. AI-assisted tools are well-suited to support colposcopy evaluation and guide cervical biopsies [65,80]. Studies suggest that AI diagnostic approaches could support or even potentially replace conventional colposcopy by enabling more objective and standardized sampling of suspicious tissue specimens. This could reduce the number of missed high-grade lesions and ultimately lower cervical cancer incidence, particularly in low-resource settings where access to colposcopy is limited, by providing a cost-effective alternative to colposcopy [81]. Research also indicates that AI could assist less experienced clinicians in deciding whether to perform a cervical biopsy and where to target it [66,80].

Large studies using AI systems for grading colposcopic impressions have reported greater consistency with pathology findings compared to human interpretations. AI systems have also demonstrated greater accuracy in predicting biopsy sites. For instance, Cho et al. reported an AUC of 0.947 for distinguishing high-grade lesions requiring biopsy, outperforming experienced colposcopists in binary classification [65]. The reported sensitivity and specificity of AI in colposcopy for detecting CIN2+ lesions vary widely, ranging from 71.9 to 98.22% and 51.8 to 96.2%, respectively. The overall diagnostic accuracy ranges from 40.5% to 98.3%, depending on the image quality, training data, and model architecture [80]. Some models can also generate attention heatmaps that highlight suspicious regions, offering real-time visual guidance for lesion localization and targeted biopsy [65,82].

## 4. Performance of AI in Cervical Cytology

This section provides a structured synthesis of diagnostic performance metrics reported across various AI applications in cervical cancer screening. Building on the specific use cases outlined in the above section, we herein compare the sensitivity, specificity, and accuracy rates across cytology, HPV-based triage, and colposcopy studies, highlighting the strengths and limitations of AI performance in each domain (Table 2). It is important to note that the studies summarized here vary considerably in methodological quality, dataset size, study design (retrospective vs. prospective), and risk of bias. Many studies are single-center and retrospective, with limited external validation cohorts, which constrains the generalizability of reported performance metrics. Readers are encouraged to consider these limitations when interpreting the figures presented, and future systematic reviews with formal quality appraisal tools (e.g., QUADAS-2) are warranted to provide more definitive comparative assessments.

In primary cervical cytology, AI-assisted reading has shown comparable or superior diagnostic performance compared to expert cytologists, highlighting the capacity of AI to match or even exceed human pathologists in controlled settings. The reported accuracy values for distinguishing between normal and cancerous Pap smears range from 80% to 100%, with lesion-specific detection (e.g., CIN1–3 or adenocarcinoma in situ) varying from 67% to 98.3%, depending on the model architecture, dataset size, and labeling consistency [80]. In one study, AI reviewed slides up to 380 times faster than an average human pathologist, highlighting its potential to reduce turnaround times in high-volume settings [76]. An observational study reported detection rates for CIN 2 and CIN 3+ of 92.6% and 96.1%, respectively, which were significantly higher than those of manual reading [83]. Another large population-based cohort study showed a high total agreement rate (94.7%) between AI and manual reading, with a 5.8% increase in sensitivity compared to manual reading [83]. These results suggest that AI systems not only support but also improve primary screening accuracy, especially when deployed at scale.

AI-based triage systems have shown promising results for HPV-positive women in reducing unnecessary colposcopy referrals without compromising diagnostic accuracy [68,79]. Wentzensen et al. developed a two-stage model to interpret dual-stained cytology (p16/Ki67), achieving 88.1% sensitivity and 61.5% specificity for CIN3+, while reducing referral rates compared to Pap cytology by nearly 20 percentage points [68]. Similarly, Xue et al. demonstrated improved sensitivity and specificity when junior cytopathologists were assisted by AI, compared to manual interpretation [67]. Another study reported that an AI-assisted LBC model achieved 94.6% sensitivity and 89.0% specificity, outperforming both human readers and HPV16/18 genotyping in a prospective multicenter setting [49]. These findings support the role of AI as an efficient and scalable triage tool in HPV-based screening programs.

The diagnosis of ASC-US has traditionally required further triage to identify women at risk of underlying CIN2+ lesions. Within this cytology-based triage context, Tao et al. developed a deep learning model to stratify ASC-US smears for CIN2+ detection [64]. In this restricted ASC-US subgroup, the AI model showed higher sensitivity (92.9% vs. 89.3%) and specificity (49.7% vs. 34.3%) than HPV testing for immediate CIN2+ triage [87]. Given the high prevalence of HPV and the often indeterminate nature of ASC-US, AI triage may reduce over-referral and overtreatment while preserving clinical safety, especially in populations with high hrHPV prevalence and limited cytological specificity [78]. Studies have reported sensitivities of 71.9–98.2% and specificities of 51.8–96.2% across various AI-assisted colposcopy systems, with the potential to support less experienced clinicians and improve biopsy decision-making [66,80,88].

## 5. Benefits and Challenges of Artificial Intelligence in Cervical Cytology

Taking everything into consideration, the advantages of AI-driven algorithms in cervical cytology include a significant reduction in sample analysis time, high sensitivity, and excellent scalability, which ensures that the derivable benefits are proportional to the scale of implementation. Moreover, faced with a growing demand for diagnostics and a concurrent shortage of pathologists [89] and highly skilled professionals, the application of these advanced AI tools serves to minimize the risk of diagnostic error [63,80]. In contrast to manual cytology interpretation, which is known to be subjective, to be dependent on the observer’s experience, and to show intra- and inter-observer variability, AI offers an automated, objective, and constant method to support cytopathologists and cytotechnologists. The standardization directly addresses a key limitation of the traditional approach [49,90,91]. In resource-limited areas of the world, where access to medical care is limited by financial and logistical factors, AI could provide a cost-effective point-of-care screening option. Holmström et al. successfully explored such an approach in a study conducted in rural Kenya [75].

However, the implementation of AI in cytology requires the prior deployment of digital cytology infrastructure [92]. Scanners, digital storage, and adequate computational resources are essential, all of which necessitate substantial initial investment. Future development should prioritize making AI technologies more cost-effective, robust, and accessible, thereby facilitating their implementation in resource-limited settings [93]. In addition, extensive databases can be leveraged to train new generations of cytopathologists and provide opportunities for continuous professional development, reducing subjectivity and enhancing diagnostic precision [15,80,94].

Despite the potential, developers of algorithms that support pathologists in everyday cervical cancer routines face several challenges. Achieving satisfying results requires large, open, standardized, and diverse datasets, including ground truth diagnoses. Such resources remain scarce, complicating the creation of robust models [95]. Performance can also be affected by variability in cytological features, for example, differences related to the type of LBC preservative solution used [84]. Continued research is necessary to improve AI algorithms for complex tasks such as segmenting clustered and overlapping cells in cytology images [41].

Many AI models have only been evaluated retrospectively, often using surrogate endpoints outside real-world clinical settings. Prospective clinical trials are urgently needed to validate findings and highlight differences between models [18]. Independent validation is equally critical. Of nearly 300 AI-enabled medical devices approved by the FDA, only a small fraction has undergone evaluation in prospective randomized controlled trials, where 81% achieved their primary endpoints; in cervical cytology, the reported accuracies reached 94% for Pap smears and 90% for the ThinPrep cytologic test [18,63]. To improve transparency and reproducibility, standardized reporting guidelines such as the CONSORT-AI extension have been introduced. This framework outlines 25 extended items across key sections of a trial report, including title, abstract, introduction, methods, randomization, results, discussion, and other relevant information [96]. Furthermore, systematic reviews, meta-analyses, and head-to-head comparative studies are needed to objectively evaluate the relative effectiveness of different AI techniques [97].

Another challenge lies in the regulatory difference in certification requirements across regions. FDA approval is centralized and focuses heavily on direct data evaluation and clinical trial evidence, a process that is more time-consuming and costly. In contrast, European certification under the IVDR is decentralized, with greater emphasis on regulatory compliance, data security, and quality management; for lower-risk categories, the process is considerably simplified. There is currently only one FDA-approved tool available for cervical cytology (Hologic Genius), but at least three were recently CE-IVD (whether IVDR or IVDD)-approved [98,99].

The increasing adoption of AI in medicine has sparked debate over the ethical implications of its use. One of the greatest dilemmas concerns responsibility for misdiagnosis—specifically, whether liability lies with the manufacturer, physician, or medical institution. Many authors argue that moral responsibility can only be assigned to humans if they make the final decision regarding diagnosis [100,101]. This dilemma is compounded by the nature of many AI algorithms, which work as a “black box”—tools that do not explain how decisions are made [102]. Ethical principles also dictate that patients have the right to be informed, to refuse testing, and, above all, to provide informed consent. This raises questions about whether patients should be allowed to refuse the use of AI in diagnostic workflows, and whether consent can truly be considered informed when the rationale for the diagnosis remains unknown [103].

Successful clinical integration will depend not only on technological advancements but also on targeted educational initiatives. Incorporating AI-focused training into pathology residency and cytotechnology curricula will be essential for preparing the next generation of practitioners [104]. Sustained collaboration among AI developers, clinicians, and regulatory bodies is equally important to streamline approval pathways and ensure safe, effective deployment in routine practice [18].

### Strengths and Limitations

A major strength of this review is its broad overview of artificial intelligence applications across the cervical cytology pathway, including automated slide screening, liquid-based cytology, ASC-US triage, HPV-positive triage, and colposcopy. The review also integrates technical, clinical, regulatory, and ethical perspectives, which is important for understanding the real-world potential of AI in cervical cancer prevention. By summarizing both commercially available systems and research-stage models, the manuscript highlights the rapid development of this field and its possible relevance for routine practice, particularly in settings with limited human resources.

This review also has several limitations. First, it is a narrative review and not a systematic review, and the included studies were not identified or appraised using a predefined systematic methodology. Second, the cited literature is highly heterogeneous with respect to study design, target population, sample preparation, scanner platform, AI architecture, reference standard, and clinical endpoint. Some studies were conducted in referral populations, enriched datasets, or retrospective research settings rather than routine screening populations, which may overestimate performance and limit generalizability. In addition, some studies evaluated AI in primary cytology screening, whereas others addressed the triage of HPV-positive women, ASC-US cases, dual-stain cytology, or colposcopy, making direct comparison across studies difficult. Reported performance metrics are therefore not always directly comparable and should be interpreted in the context of the specific clinical setting and intended use. Finally, the relevance of some older cytology-based triage studies to modern HPV-primary screening programs may be limited. These considerations should be taken into account when interpreting the published evidence and judging the readiness of AI systems for wider clinical implementation.

## 6. Conclusions

Artificial intelligence has considerable potential to improve cervical cytology and related triage workflows by increasing efficiency, standardization, and diagnostic consistency. Recent studies suggest that AI-assisted systems can support abnormal-cell detection, slide classification, risk stratification, and selected triage tasks, including the assessment of equivocal cytology, HPV-positive women, dual-stain cytology, and colposcopic images. These applications may help reduce the workload of cytotechnologists and cytopathologists and may be particularly valuable in settings with limited human resources and growing demands for screening and diagnostic services.

At the same time, the published evidence should be interpreted with caution. The reported performance varies substantially across studies because of differences in study design, target population, sample preparation, scanner platform, AI model, reference standard, and clinical endpoint. Many studies have been conducted using retrospective or enriched datasets, in referral populations, or in specific triage settings rather than in routine primary screening populations. As a result, promising performance metrics cannot automatically be generalized to real-world screening practice, particularly in modern HPV-primary screening programs, where cytology increasingly serves as a triage test rather than the primary screening method.

The successful clinical implementation of AI in cervical cytology will therefore require more than technical performance alone. Robust prospective validation, transparent reporting, clinically meaningful endpoints, regulatory approval, cost-effective digital infrastructure, and careful integration into existing workflows are all essential. Ethical and legal issues, including accountability, transparency, and informed consent, must also be addressed.

## Figures and Tables

**Table 1:** Examples of AI platforms and clinical applications in cervical cancer screening and diagnosis.

Application	Platform	Function
Automated and assisted slide screening	Genius Digital Diagnostics, CytoProcessor, CytoSiA, Techcyte	Identifying abnormal areas for cytopathologist review; flagging slides as cancerous or non-cancerous [22,63]
AI-enhanced microscopes	AI microscope equipped with an augmented reality technique	Real-time overlay of AI results onto field of view [10]
ASC-US risk stratification	Custom DL models	Stratifying ASC-US cytology cases for CIN2+ risk [64]
Liquid-based cytology	YOLOv5 dCNN, custom hybrid CNN-RNN, DeepCyto	WSI-based classification of neoplastic vs. non-neoplastic [45,49]
Colposcopy	Multi-task CNN, ResNet-152, ResNet-50, XGBoost, ResNet18	Assisting lesion grading and biopsy targeting [65,66]
HPV-based screening	CNN4 + Inception-v3; EfficientNet, ResNer-50 variants	Multimodal risk stratification for CIN2+ (DL models, HPV tests, clinical assessment) [67,68]

ASC-US—atypical squamous cells of undetermined significance AI—artificial intelligence; DL—deep learning; CNN—convolutional neural network; dCNN—deep convolutional neural network; RNN—recurrent neural network; WSI—whole-slide images; HPV—human papilloma virus; CIN—cervical intraepithelial neoplasia.

**Table 2:** Summary of diagnostic performance metrics for AI applications across cervical cancer screening domains.

Domain	Sensitivity	Specificity	AUC	Performance
Automated and assisted cytology	95% (vs. Pap) [63]	94% (vs. Pap) [63]	Up to 0.99 [76]	AI showed a detection rate of 92.6% for CIN2+ and 96.1% for CIN3+ 96.1%; 95% agreement between AI and manual reading [22,83]. Relative sensitivity vs. experts: 1.01; relative specificity vs. experts: 1.26 [83]
AI-enhanced microscopes	95%	0.9	0.81	Improved sensitivity compared to manual reading: LSIL+ from 0.860 to 0.950; ASC-H from 0.817 to 0.910; improved intraobserver agreement: binary from 0.649 to 0.706, multiclass from 0.720 to 0.798, ASC-US from 0.581 to 0.637 [10].
ASC-US risk stratification	92.9%	49.7%	0.79	AI outperformed hrHPV in CIN2+ triage with no added cost (sensitivity 92.9% vs. 89.3%; specificity 49.7% vs. 34.3%; AUC 0.79 vs. 0.61); relevant in populations with high HPV prevalence and high false-positive rates [64].
Liquid-based Cytology	87.8–99.1%	83.1–99.6%	0.85–0.993	Best performance observed when AI-assisted cytopathologist review (up to 99.1% sensitivity, 99.6% specificity, AUC 0.993) [49]; good performance on multi-cell images, but limited generalizability to overlapping AI-assisted [44]; high intraobserver variability in NILM cases [45]; preservative type affects AI detection rates and should be accounted for [84].
Colposcopy	71.9–98.2%	51.8–96.2%	up to 0.947	Variable diagnostic values for CIN2+ detection [65,81,82,85,86]; AI was superior to colposcopists in grading agreement and biopsy site prediction [66]; mean AUC to determine the need for biopsy was 0.947 [65]; especially useful in low-resource settings [81].
HPV-based screening	85.7–87%	45.6–84%	0.74–0.96	AI-assisted reading increased sensitivity (from 65.7% to 85.7%) and specificity (from 73.7% to 84.0%), and reduced review time (from 218 s to 30 s) compared to manual reading in detecting CIN2+ [67]; lower positivity, similar sensitivity, and higher specificity than cytologists in detecting CIN3+ [68]; AI reduced colposcopy referrals by 10–20 percentage points [68,79]; applicable in a low-resource setting [78].

Unless otherwise stated, diagnostic values refer to detection of CIN2+. Important note: Performance metrics presented in this table are derived from heterogeneous studies with differing datasets, endpoints, patient populations, and methodological designs. Direct numerical comparisons across rows or domains should be interpreted with caution, as these values are not derived from head-to-head comparative evaluations. Differences in sensitivity and specificity may reflect variations in study design, prevalence, and reference standards rather than true differences in AI system performance. AI—artificial intelligence; NILM—negative for intraepithelial lesion or malignancy; AUC—area under the receiver operating characteristic curve; Pap—test Papanicolaou; ASC-H—atypical squamous cells; hrHPV—high-risk human papillomavirus.

## Data Availability

No new data were created or analyzed in this study.
